# Intercellular Adhesion Molecule-1 as Target for CAR-T-Cell Therapy of Triple-Negative Breast Cancer

**DOI:** 10.3389/fimmu.2020.573823

**Published:** 2020-09-23

**Authors:** Heng Wei, Zeng Wang, Yi Kuang, Zhiguo Wu, Shasha Zhao, Zongliang Zhang, Hexian Li, Meijun Zheng, Nan Zhang, Cheng Long, Wenhao Guo, Chunlai Nie, Hui Yang, Aiping Tong

**Affiliations:** ^1^State Key Laboratory of Biotherapy and Cancer Center, West China Hospital, West China Medical School, Sichuan University, Chengdu, China; ^2^Department of Otolaryngology, Head and Neck Surgery, West China Hospital, Sichuan University, Chengdu, China; ^3^West China-Frontier Pharma Tech Co., Ltd. (WCFP), Chengdu, China; ^4^Department of Orthopaedics, West China Hospital, Sichuan University, Chengdu, China

**Keywords:** triple-negative breast cancer, intercellular adhesion molecule-1, chimeric antigen receptor T cells, immunotherapy, single chain variable fragment

## Abstract

Triple-negative breast cancer (TNBC) comprises lethal malignancies with limited treatment options. Chimeric antigen receptor T (CAR-T) cell therapy is an effective immunotherapeutic strategy that has demonstrated unprecedented efficacy in the treatment of hematological malignancies but has shown limited success in the management of some solid tumors. Many malignant tumors are related to increased expression of intercellular adhesion molecule-1 (ICAM1), providing a rationale for ICAM1-specific immunotherapies for the treatment of cancer. Here, we validated the expression of ICAM1 in TNBC tissues. Subsequently, we generated a phage-displayed single-chain variable fragment (scFv) library using splenocytes from ICAM1-immunized mice and selected a novel ICAM1-specific scFv, mG2-scFv. Using mG2-scFv as the extracellular antigen binding domain, we constructed ICAM1-specific CAR-T cells and demonstrated the robust and specific killing of TNBC cell lines *in vitro*. Most importantly, in the TNBC mouse model, ICAM1-specific CAR-T cells significantly reduced the growth of the TNBC tumor, resulting in long-term remission and improved survival. Together, these results indicated that ICAM1-specific CAR-T cells have high therapeutic potential against ICAM1-positive TNBC tumors.

## Introduction

Triple-negative breast cancer (TNBC) is a type of breast cancer with high invasiveness and recurrence rate, accounting for 15–20% of all confirmed breast cancer cases (1). Since TNBC lacks the expression of the human epidermal growth factor receptor 2 (HER2), estrogen receptor (ER), and progesterone receptor (PR) (2), hormone therapy and HER2−targeted therapy are ineffective toward the treatment of TNBC (3). At present, the commonly used methods for treating TNBC are mainly surgery, chemotherapy, and radiotherapy (4). However, these treatments usually lead to pancytopenia and other adverse events such as nausea and diarrhea (5). Although response rates to PD-1/L1 monoclonal antibodies are higher in TNBC than in other breast cancer subtypes, the efficacy is still low, with monotherapy response rates ranging approximately between 5 and 23% (6). Recently, some targeted drugs have been put into preclinical and clinical trials for TNBC, but most of them have not achieved satisfactory therapeutic results (7, 8). Therefore, new and powerful therapies are urgently needed for TNBC patients.

Chimeric antigen receptors (CARs) are synthetic receptors that when engineered into T cells can redirect them to tumor cell surface antigens, get activated to become cytolytic, and eventually eliminate targeted tumor cells (9, 10). Various CARs can be generated by fusing the antigen-specific variable single-chain fragments with T cell activating and co-stimulatory signaling domains (11, 12). Chimeric antigen receptor T (CAR-T) immunotherapy is an effective immunotherapy strategy and plays an important role in cancer treatment. Specifically, CD19-targeted CAR-T cells have yielded encouraging results in the treatment of B cell malignancies and have been approved by the US Food and Drug Administration (13, 14). Despite their success in treating hematologic malignancies, the therapeutic effects of CAR-T cells on solid tumors are unsatisfied. One of the main reasons is the lack of specific target cell surface antigens (15). Thus, specific tumor target antigens are critical for engineering CAR-T cells.

Intercellular adhesion molecule-1 (ICAM1) is a cell surface transmembrane glycoprotein receptor belonging to the immunoglobulin superfamily. Intercellular adhesion molecule-1 plays important roles in cell adhesion, cell signaling, and transendothelial migration of leukocytes to sites of inflammation (16). Overexpression of ICAM1 had been reported in several malignancies, including renal cell carcinoma, pancreatic cancer, and lung cancer (17–19). Moreover, some tumors with high levels of ICAM1 were correlated with metastasis and poor prognosis (20). Previous studies have shown that inhibition of ICAM1 decreases metastasis in melanoma and lung cancer (21, 22). Studies have demonstrated that the expression of ICAM1 in TNBC is higher than that in other types of breast cancer (23, 24), and it has been speculated that ICAM1 can serve as a therapy target for TNBC. Recently, ICAM1 as a therapeutic target for TNBC treatment achieved satisfactory results (25, 26). Accordingly, we speculated that ICAM1 is one of the favorable targets for engineering CAR-T cells.

In this study, we generated ICAM1-specific CAR-T cells and performed phenotypic analysis of these T cells. Our results from *in vitro* and *in vivo* experiments revealed that ICAM1-specific CAR-T cells exerted high antigen specificity and strong antitumor effects against TNBC.

## Materials and Methods

### Cell Lines and Mice

The HEK-293T, A-431, Hela, MDA-MB-231, MDA-MB-468, SKBR3, and MCF10a cell lines were all obtained originally from the American Type Culture Collection (ATCC). HEK-293T, A-431, Hela, MDA-MB-231, MCF-7, and MDA-MB-468 were cultured in Dulbecco’s Modified Eagle Medium (DMEM; Gibco, Life Technologies) and SKBR3 in Roswell Park Memorial Institute (RPMI)-1640 (Gibco, Life Technologies). These cells were cultured in medium containing 10% fetal calf serum and 1.0 mmol/L penicillin–streptomycin combination (Hyclone). MCF10a cells were cultured in DMEM/F12 (Gibco, Life Technologies), supplemented with 5% horse serum (Gibco, Life Technologies), 20 ng/ml EGF (Sigma-Aldrich), 0.5 μg/mL hydrocortisone (Sigma-Aldrich), 100 ng/mL cholera toxin (Sigma-Aldrich), and 10 μg/mL insulin (Sigma-Aldrich). All cell lines were cultured in a humidified incubator with 5% CO_2_ at 37°C. In this study we used six-week-old female NSG mice (NOD.Cg-Prkdcscid Il2rgtm1Wjl/SzJ), purchased from the Model Animal Resource Information Platform of Nanjing University, P. R. China and maintained under pathogen-free conditions.

### Bioinformatic Analysis

UALCAN^1^ is an interactive web portal that utilizes publicly available RNA-sequence and patient clinical data generated by the TCGA consortium. The expression of ICAM1 in different BRCA (breast invasive carcinoma) subtypes and normal samples were analyzed using the online web server. Expression of ICAM1 at the mRNA level in 56 breast cancer cell lines were analyzed using the CCLE database^2^.

### Immunization and Library Construction

Seven-week-old female BALB/c mice were first immunized with hICAM1 extracellular domain (ECD)-Fc mixed with an equal volume of Freund’s complete adjuvant (Sigma-Aldrich), and then continued to accept immunization with Freund’s incomplete adjuvant (Sigma-Aldrich) once every 10 days. After the fifth immunization, splenocytes were isolated from mice, and a phage display library containing the genes coding for the single-chain variable fragment (scFv) was then generated according to established methods (27).

### ICAM1-scFv Selection, Expression, and Purification

Intercellular adhesion molecule-1-scFv were screened through phage display and ELISA, according to previously described procedure (28). The scFv fragments were amplified and subcloned into Pvax-CMV-β-globin vector. The recombinant vector was transfected into HEK-293F cells and the scFv-Fc protein was purified using protein A and nickel-nitrilotriacetic acid (Ni-NTA) affinity chromatography (GE Healthcare). Finally, the purity of scFv-Fc was detected by SDS-PAGE.

### Knockout of ICAM1 in MDA-MB-231 Cells

Knockout of ICAM1 in the MDA-MB-231 cells were generated through the CRISPR-Cas9 system. gRNAs were designed using the online server CHOPCHOP^3^ and inserted into lentiCRISPR_v2 (Addgene; #52961). Lentiviral particles were transduced into MDA-MB-231 cells when the cells grew up to 30–50% confluence. Twenty-four hours after transduction, transfected cells were selected with puromycin. The knockout effect was verified by sequencing, western blotting, and immunofluorescence staining. DNA isolation and sequencing was performed according to previous publish method (29). The forward primer flanking the ICAM1 target was 5′-GCAAGGTCCACTTCACCAGACACC-3′, and the reverse primer was 5′- ACTCAGCAGCCTAGGTCACATACG-3′.

### Western Blotting

Western blotting was performed as described previously (29). In brief, cell lysates were prepared using RIPA lysis buffer (Beyotime) supplemented with protease inhibitor cocktail (Beyotime). Mouse monoclonal anti-ICAM1 antibody (Huabio) and mouse monoclonal anti-β-actin antibody (Beyotime) were diluted in 1:2,000. Detection was performed with HRP-conjugated rabbit anti-mouse IgG antibody (Beyotime) diluted in 1:3,000. Targeted protein bands were detected by enhanced chemiluminescence and digital imaging (Clinx Science Instruments, Chemiscope 5300).

### Affinity Determination

The kinetics of mG2-scFv binding to human recombinant hICAM1 antigen was determined by Biacore X100 instrument (GE Healthcare), according to a previously published procedure (30).

### Lentivirus Production

To produce lentivirus particles, HEK-293FT cells cultured in DMEM were co-transfected with the target plasmid together with two packaging plasmids psPAX2 and pMD.2G at a ratio of 4:3:1 using Polyethylenimine (PEI, Sigma). Supernatants were collected at 24 and 48 h after transfection, concentrated by ultracentrifugation for 2 h at ×20,000 *g*. The viruses were resuspended in RPMI-1640 and stored at –80°C until ready to use for experiments. All lentiviruses used in the experiments were from concentrated stocks.

### T Cell Transduction

Intercellular adhesion molecule-1-specific and CD19-specific CARs were constructed using a lentiviral vector encoding CD8α signal peptide, anti-ICAM1/CD19-scFv, Myc-tag, CD8α transmembrane domain, the cytoplasmic domain of human CD28, 4-1BB, and CD3ζ. CD19-specific CAR (control CAR) was used as control.

Peripheral blood mononuclear cells were isolated from healthy donors using density gradient centrifugation and grown accordingly to the previously described culture method (31).

### Flow Cytometry

The antibodies used to identify the phenotype of CAR-T cells including anti-Human CD3 (APC-Cy7), anti-Human CD4 (PE), anti-Human CD45RO (PE-Cy7), anti-Human CD62L (FITC), and anti-Human CD8 (FITC) antibodies, as well as corresponding mouse IgG controls and anti-CD54 (FITC), were all purchased from BioLegend. ICAM1 expression levels on the surface of human tumor cells and ICAM1-CAR transduction efficiency were evaluated *via* purified ICAM1-scFv-Fc and ICAM1 ECD-Fc, respectively. The transduction efficiency of control CAR-T (anti-CD19) was evaluated *via* Myc-tag mouse mAbs staining (Cell Signaling Technologies). Anti-mouse IgG-FITC (BioLegend) was used to label the Fc of ICAM1 ECD-Fc. Fluorescence was assessed using a BD Fortessa flow cytometer and analyzed using FlowJo 10.6.0 software.

### Cytotoxicity Assays

Chimeric antigen receptor T (CAR-T) cytotoxicity against target cells was assessed using the *in vitro* ^51^Cr (PerkinElmer) release assay. 1 × 10^4^ target cells labeled with sodium chromate (Na2^51^CrO4) were incubated with effector cells at an E:T ratio of 16:1, 8:1, 4:1, and 2:1 for 4 h at 37°C in 5% CO_2_. The supernatant was harvested and ^51^Cr release into media was measured by gamma counter. Finally, the cytotoxicity of T cells was calculated according to the following formula: 100% × [(test release - spontaneous release)/(maximum release - spontaneous release)], where test release represents the ^51^Cr release from the target cells co-incubated with T cells, spontaneous is the release from target cells without T cells, and maximum release is from target cells lysis.

### Cytokine Release Assays

For *in vitro* experiments, 1 × 10^4^ cells were seeded in a 96-well plate under standard cell-culture conditions. The antigen-specific cytokine release was performed by co-culturing CAR-T cells with target cells at E:T of 4:1 ratio. After 24 h of co-culture, supernatants were collected. The IL-2, IFN-γ and TNFα were measured by ELISA assay kit (BioLegend). For *in vivo* experiments, 24 h after the injection of T cells, 100 μL of peripheral blood was collected from mice. The IL-2, IL-6, IFN-γ, and TNFα were analyzed by ELISA assays. All ELISAs were performed following the operation manual.

### Immunohistochemistry Assay

To assess the expression of CD3 on human T cells and ICAM1 in xenograft models, tumor samples were fixed with 4% paraformaldehyde, embedded in paraffin blocks, and then micro-dissected into several sections. Sections were incubated at 65°C for 1 h to retrieve antigenicity, blocked for 1 h at room temperature followed by incubation with anti-ICAM1 and anti-CD3 mouse monoclonal antibody (BioLegend, 1:100) at 4°C, overnight. All procedures followed the manufacturer’s protocol. Saturation and intensity of immunostained cells was evaluated over four visual fields under a light microscope. Staining intensity was scored as follows: 0 was negative, 1 was weak positive (+), 2 was positive (++), and 3 was strong positive (+++). The percentage of cells stained was estimated as follows: Intensity score = (1 × weak positive staining% + 2 × positive staining% + 3 × strong positive staining%) × 100.

### Immunofluorescence Staining

Cells were seeded in 24-well plates under standard cell-culture conditions. After 24 h, medium was removed. Cells were rinsed in phosphate-buffered saline (PBS) and fixed for 15 min in 4% paraformaldehyde, followed by washing with PBS. After blocking with 1% BSA, cells were stained with the primary antibody (ICAM1-scFv-Fc) for 1 h at 4°C. Next, samples were stained with a FITC-conjugated secondary antibody (Proteintech) for another 1 h, followed by washing with PBS. 4′,6-diamidino-2-phenylindole (Beyotime) was used to stain the cell nucleus. Images were acquired using a confocal microscope (Zeiss 880).

### Xenograft Mouse Models and Live Cell Imaging Assays

To establish xenograft mouse models, six-week-old female NSG mice were injected subcutaneously into the right flank with 6 × 10^6^ MDA-MB-231-luc cells or SKBR3-luc cells. At day 7 after injection, 1 × 10^7^ control CAR-T cells, 1 × 10^7^ ICAM1-specific cells or 100 μL of PBS were administered *via* the caudal vein. For the SKBR3 group, only 100 μL of PBS or 1 × 10^7^ ICAM1-specific cells were injected. Mice were intravenously treated with 100U IL-2. For antitumor efficacy analyses, tumor growth or killing in live mice was assessed by whole-body imaging using an IVIS system (Caliper Life Sciences, Hopkinton, MA, United States). Measurements were performed once a week.

## Statistical Analysis

All statistical analyses were performed using GraphPad Prism 7.0. *In vitro* experiments were repeated at least three times. Data were presented as mean ± SD or mean ± SEM. For Kaplan–Meier overall survival analysis, a log-rank test was used to compare each of the arms. For comparison between two groups, a two-tailed t-test was used. Differences with a *p*-value <0.05 were considered to be statistically significant.

### Ethical Approval

All applicable international, national, and/or institutional guidelines for the care and use of animals were followed. All procedures performed in studies involving animals were in accordance with the ethical standards of the institution and approved by the Biomedical Ethics Committee of West China Hospital of Sichuan University.

## Results

### ICAM1 Expression in TNBC

The mRNA and protein expression levels of ICAM1 were analyzed using the UALCAN web server. The expression levels of mRNA for ICAM1 were higher in breast cancer tissues compared with normal tissues (Supplementary Figure 1A). More importantly, compared with other subtypes of breast cancer, the expression of ICAM1 in TNBC was significantly increased (Supplementary Figures 1B,C). We next analyzed the ICAM1 RNA sequencing expression data in 29 TNBC cell lines and 27 non-TNBC cell lines using the CCLE web server. As a result, ICAM1 mRNA levels were higher in the TNBC cell lines than in the non-TNBC cell lines (Supplementary Figure 1D).

**FIGURE 1:**
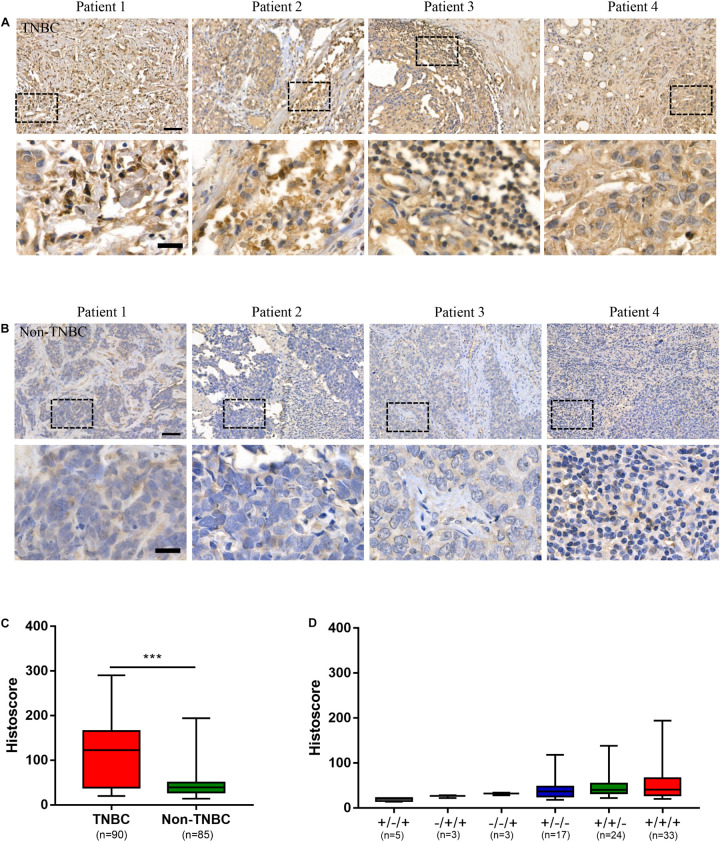
ICAM1 was upregulated in triple negative breast cancer (TNBC) tissues. **(A,B)** Microarrays of human TNBC tumors and non-TNBC tumors were stained with an anti-human ICAM1 antibody for IHC to detect the expression of ICAM1. Scale bars indicated 50 μm (top) and 20 μm (bottom). **(C,D)** ICAM1 staining intensities in different subtypes of non-TNBC (status of ER/PR/HER2: ±/+; −/+/+; −/−/+; ±/−; +/±; +/+/+) and TNBC tissues. Data are presented as a box-and-whisker plot. ***p < 0.001.

To evaluate the potential of the tumor antigen ICAM1 as an immunotherapeutic target, we performed IHC staining to detect ICAM1 protein expression in breast cancer tissue microarrays. Results showed that ICAM1 staining in TNBC tissues was stronger and present in more cells compared with non-TNBC tissues (Figures 1A,B). The ICAM1 intensity score for TNBC and non-TNBC patient samples were 117.4 ± 7.318 and 45.81 ± 3.375, respectively (Figure 1C). In addition, low or undetectable level of ICAM1 was expressed in non-TNBC across each pathological subtype, and there was no difference in the staining intensity among them (Figure 1D). A complete description of the immunohistochemical results is provided in Supplementary Table 1. This result is consistent with results from previous research (23). Altogether, these data revealed ICAM1 as a possible novel target for CAR-T cell therapy in TNBC.

### Generation and Characterization of scFv Against ICAM1

To screen for ICAM1-specific scFv, phage display bio-panning rounds were conducted. Next, the ICAM1-specific scFv was generated and named as mG2-scFv. Thereafter, mG2-scFv-Fc recombinant proteins were purified through the HEK-293F expression system, and the purity was analyzed by SDS-PAGE (the 70 kDa band corresponds to monomer and the 140 kDa band to dimer formation) (Supplementary Figure 2A). The binding affinity of mG2-scFv-Fc to ICAM1 ECD recombinant protein was measured using the Biacore X100 instrument (Supplementary Figure 2B). The affinity determination assay indicated that the KD value of mG2-scFv was 2.645E-09M.

Specific recognition of mG2-scFv was examined further in ICAM1 knockout (MDA-MB-231 ICAM1^*KO*^) cells. MDA-MB-231 ICAM1^*KO*^ cells were generated by using CRISPR/Cas9 tool. As shown, overlapping peaks were obviously observed downstream of the Cas9 cleavage site (Supplementary Figure 2C). Western blotting result show that expression of ICAM1 was significantly decreased in MDA-MB-231 ICAM1^*KO*^ cells (Supplementary Figure 2D). Next, the specific binding of mG2-scFv to ICAM1 was analyzed using IF staining. As a result, mG2-scFv specifically bound to MDA-MB-231 cells but not to the MDA-MB-231 ICAM1^*KO*^ cells (Supplementary Figure 2E). In addition, we tested the ability of mG2-scFv to cross-react with mouse ICAM1 (mICAM1) using FACS. As shown, mG2-scFv failed to recognize mICAM1 (Supplementary Figure 2F).

### Generation and Characterization of Anti-ICAM1 CAR-T Cells

**FIGURE 2:**
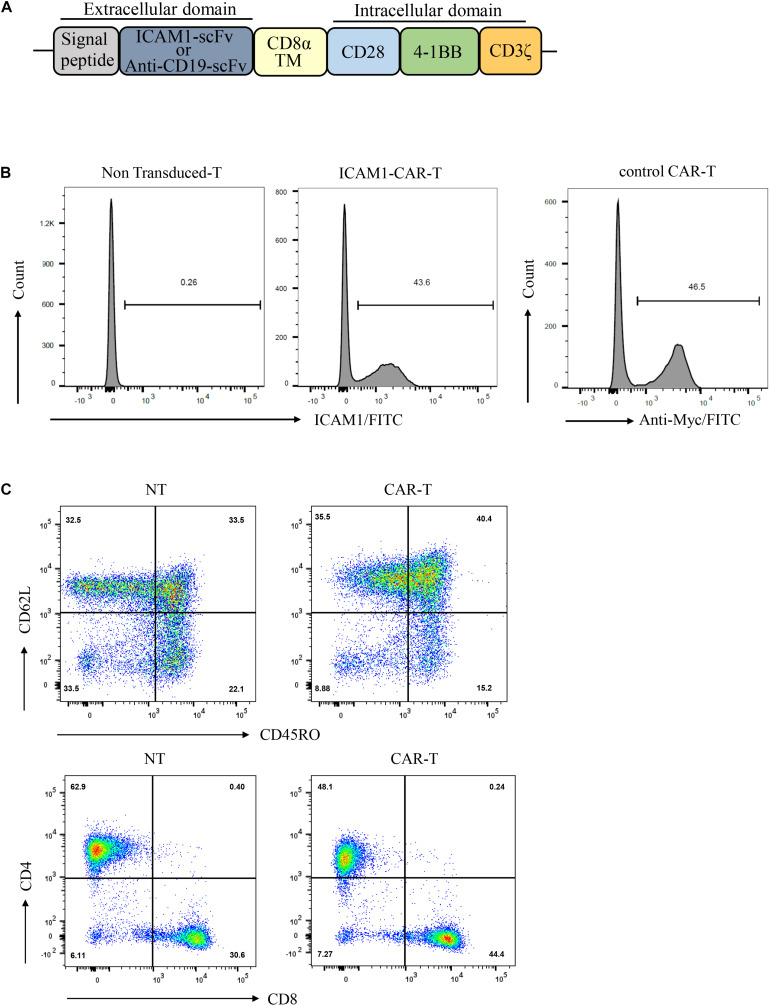
Production of ICAM1-specific CAR-T Cells. **(A)** Schematic representation of the ICAM1-specific CAR and control CAR (anti-CD19 CAR) constructs containing the following fragments: mG2-scFv/anti-CD19-scFv; CD8α transmembrane domain; intracellular signaling domains of CD28, 4-1BB, and CD3-ζ. **(B)** The ICAM1-CAR and CD19-CAR expressing human T cells were stained with antigen and then detected by FACS. **(C)** Ten days after lentivirus CAR transduction, the subsets and phenotypes of NT and ICAM1-specific CAR-T cells were analyzed by FACS, including the expression of CD4, CD8, CD45RO, and CD62L.

The CAR sequence targeted at ICAM1 was constructed based on a lentiviral vector encoding the ICAM1 binder mG2-scFv, Myc-tag, CD8αtransmembrane domain, CD28 and 4-1BB serving as co-stimulatory signals, CD3ζ mediating T-cell activation. The CD19-specific CAR (control CAR) was used as control (Figure 2A). Primary human T cells from healthy donors were transduced with either ICAM1-specific or CD19-specific CAR transgenes with similar transduction rates (unsorted with 40–50% transduction levels) (Figure 2B). After 10 days of lentivirus transduction, CAR-T cells were uniformly positive for CD8 and CD4 with a ratio of 1:1 and achieved a final CD45RO + CD62L + phenotype (Figure 2C). This result showed that these markers did not differ significantly between the CAR-T cells and non-transduced T cells.

### ICAM1-Positive TNBC Cells Are Sensitive to Anti-ICAM1 CAR-T Cells

**FIGURE 3:**
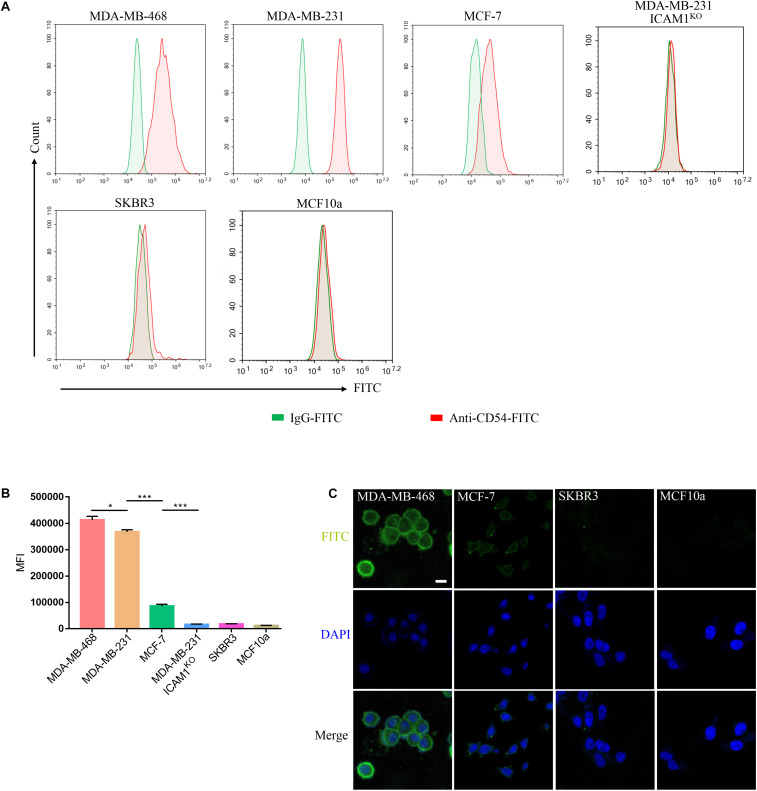
Binding of mG2-scFv to endogenous ICAM1 in breast cancer/normal cell lines. **(A)** Expression of ICAM1 in human breast cancer cells and normal breast cell were evaluated by FACS. Cells were incubated with anti-ICAM1-FITC (red) or IgG-FITC control (green). **(B)** Quantification of ICAM1 staining intensities in various breast cancer cells and normal breast cells. Unpaired two-tailed Student’s *t*-tests were applied. *p < 0.05, ***p < 0.001. **(C)** IF staining with mG2-scFv-Fc in target cells. Scale bars, 50 μm.

The surface ICAM1 expression of two human TNBC cell lines (MDA-MB-231 and MDA-MB-468), ER-positive breast cancer cell line MCF-7, MDA-MB-231 ICAM1^*KO*^ cell line, normal human mammary epithelial MCF10a, and HER2-amplified luminal breast cancer cell line SKBR3 were examined by FACS. MDA-MB-231 and MDA-MB-468 cells expressed ICAM1, whereas SKBR3, MDA-MB-231 ICAM1^*KO*^, and MCF10a did not. MCF-7 cells showed very low level of ICAM1 expression (Figure 3A). MDA-MB-231 and MDA-MB-468 exhibited a significant increase in Intercellular adhesion molecule-1 expression compared with various other subtypes of breast cancers and normal epithelium cells (Figure 3B). In order to better verify the ability of mG2-scFv to bind to endogenous ICAM1 proteins in different breast cancer cell lines, target cells were examined by IF using mG2-scFv-Fc as the primary antibody (Figure 3C). These IF results were consistent with those of the FACS analysis in Figure 3A. Similar results were observed in the other ICAM1-postive cell lines (Supplementary Figures 3A–C).

**FIGURE 4:**
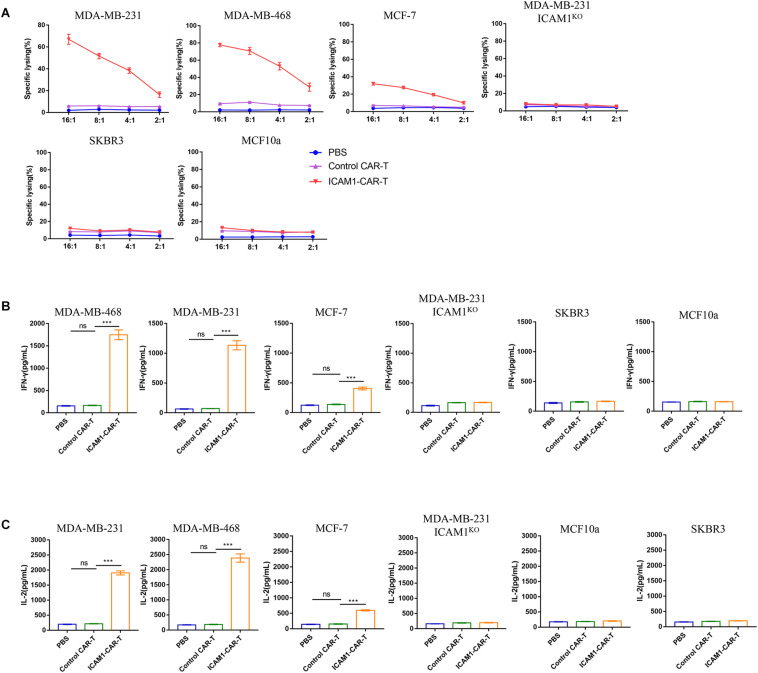
Cytokine release and cytotoxicity assay. **(A)** The lytic capacity toward target cells was analyzed at E:T ratios of 2:1, 4:1, 8:1 and 16:1 by ^51^Cr-release assay. **(B)** and **(C)** The concentrations of IL-2 and IFN-γ released by ICAM1-specific CAR-T cells, control CAR-T cells and PBS after co-culture with target cells 24 h at an E:T ratio of 4:1 are shown. Each experiment was repeated at least three times with similar results. For statistical analysis, unpaired two-tailed Student’s *t*-tests were applied. ***p < 0.001.

The specific recognizing and killing of ICAM1-positive breast cancer cells by ICAM1-specific CAR-T cells were evaluated using ^51^Cr-release cytotoxic assay. ICAM1-specific CAR-T cells killed more than 50% of MDA-MB-231 or MDA-MB-468 at an E/T ratio >4:1, whereas no cytotoxic effect was observed upon co-cultivation with MDA-MB-231 ICAM1^*KO*^, SKBR3, and MCF10a cells. However, ICAM1-specific CAR-T cells killed only 20% of MCF-7 at E/T ratio = 4:1 (Figure 4A). In addition, the ICAM1-specific CAR-T cells secreted approximately 10-fold more IFN-γ and IL-2 compared with control CAR-T cells (*p* < 0.001) when they were cocultured with MDA-MB-231 and MDA-MB-468. In contrast, ICAM1-specific CAR-T cells secreted only threefold more compared with control CAR-T cells when co-cultured with MCF-7 cells (Figures 4B,C). Similar results were obtained in the other ICAM1-postive cell lines (Supplementary Figure 4A–C).

### Antitumor Effect of Anti-ICAM1 CAR-T Cells *in vivo*

**FIGURE 5:**
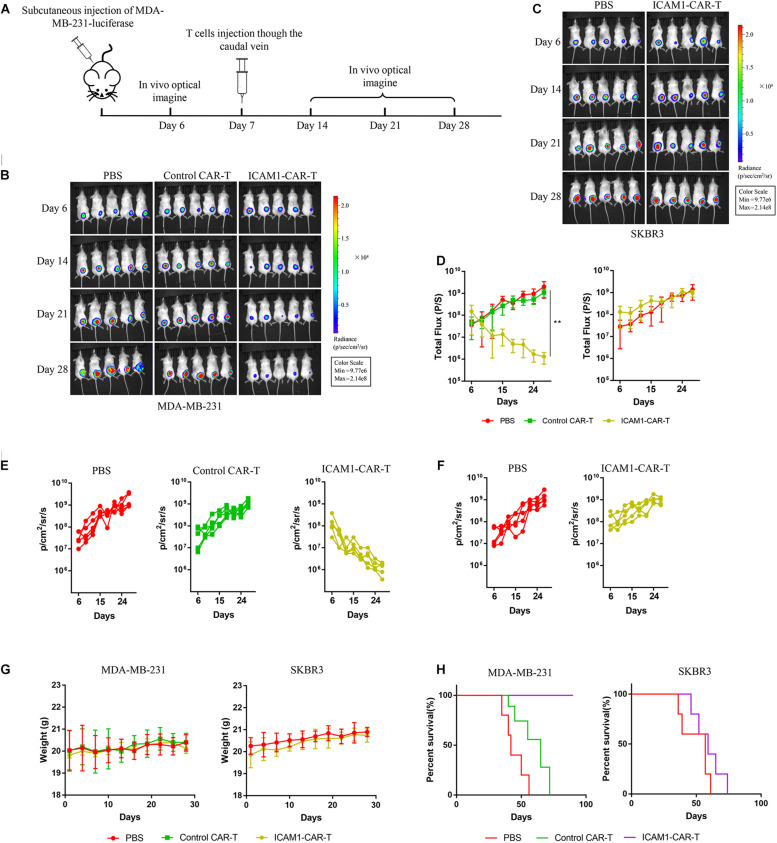
Antitumor effect of ICAM1-specific CAR-T cells in xenograft models. **(A)** Treatment scheme used in the MDA-MB-231-luc and SKBR3-luc xenograft models. **(B,C)** An image of the MDA-MB-231-luc and SKBR3-luc allograft tumor, respectively, in mice (five mice per group). **(D)** Tumor total flux data (in p/s) were calculated using Living Image software. Tumor growth rates are shown as mean values (unpaired two-tailed Student’s *t*-tests, **p < 0.01). **(E,F)** Fluorescence intensity changes of MDA-MB-231-luc and SKBR3-luc heterologous tumors in mice. **(G)** Body weight measurements during the treatment. **(H)** Overall survival of mice in each group.

**FIGURE 6:**
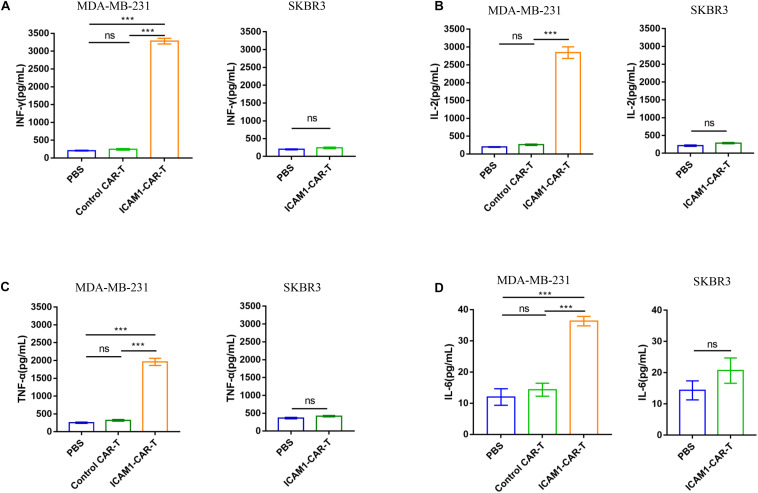
Efficacy of ICAM1-specific CAR-T cells therapy and cytokine release *in vivo*. **(A–D)** The concentrations of IFN-γ, IL-2, TNF-α, and IL-6 in the peripheral blood of mice, which was collected at 24 h after injection of T cells, were measured by ELISA. Each experiment was repeated at least three times. For statistical analysis, unpaired two-tailed Student’s t-tests were applied. ***p < 0.001.

To test the cytotoxic activity of anti-ICAM1 CAR-T *in vivo*, an NSG mouse model bearing subcutaneous MDA-MB-231-luc tumor was established (Figure 5A). The fluorescence intensity of tumor cells was monitored through *in vivo* imaging to assess tumor regression in mice. The results of mean fluorescence intensity of *in vivo* imaging showed that ICAM1-specific CAR-T cells were effective in reducing the tumor sizes in MDA-MB-231 xenograft tumors (*p* < 0.01) (Figures 5B,D,E). Next, to assess the non-specific cytotoxicity of anti-ICAM1 CAR-T cells, we established a mouse model with SKBR3-luc xenograft tumor. Our results showed that ICAM1-specific CAR-T cells had no inhibitory effect in tumor growth when compared with the PBS control group (Figures 5C,D,F). In addition, no significant changes in mouse body weight were observed during treatment (Figure 5G). We further found that this potent antitumor effect of ICAM1-specific CAR-T cells led to significant survival benefits compared with the PBS and control CAR-T groups (Figure 5H). Furthermore, we observed an approximate 3- to 16-fold increase in the concentration of cytokines (IL-2, IL-6, TNF-α, and IFN-γ) in the serum of MDA-MB-231 xenograft in ICAM1-specific treatment compared with PBS and control CAR-T. No significant increase of cytokines was observed in the SKBR3 xenograft (Figures 6A–D). These results indicate that ICAM1-specific CAR-T cells had significant antitumor effects on TNBC.

### Infiltration of Anti-ICAM1 CAR-T Cells and Expression of ICAM1

**FIGURE 7:**
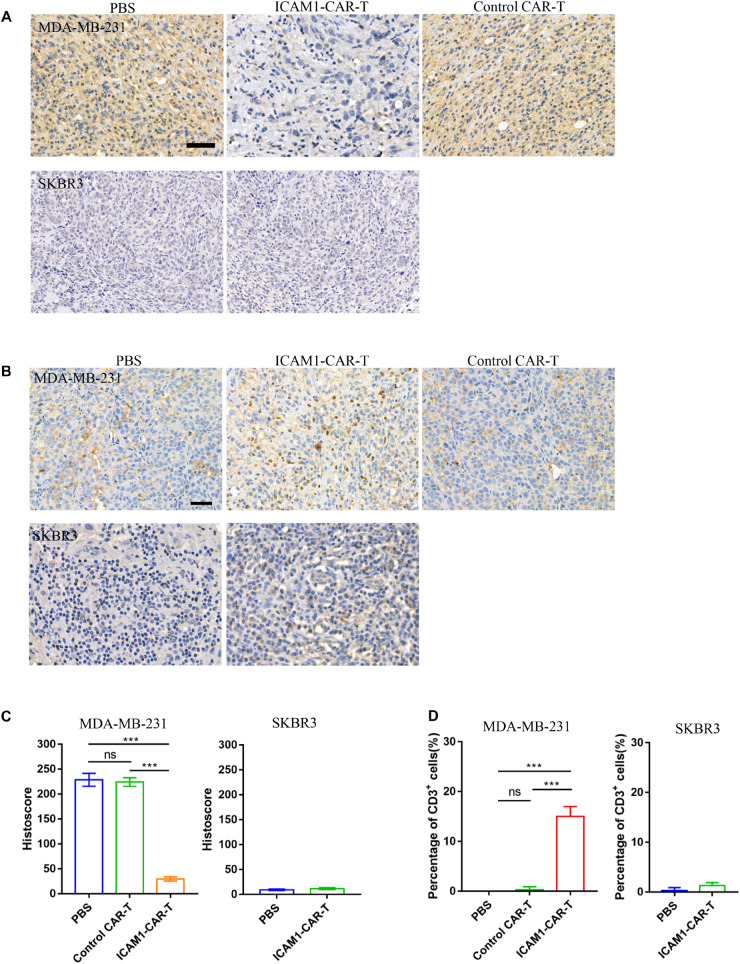
Immunohistochemistry of CD3 and ICAM1 in mouse tumor tissue. **(A)** Representative images of ICAM1 IHC staining in tumor sections after treatment. Scale bars = 50 μm. **(B)** Detection of CD3 on tumor tissue from the MDA-MB-231 and SKBR3 xenograft mouse models. **(C)** ICAM1 staining intensities in all treatment groups from the MDA-MB-231 and SKBR3 xenograft mouse models. **(D)** Determination of the number of CD3-positive cells by cell counting. Each datum represents three independent experiments (bar value represents the dispersion degree, ***p < 0.001).

We investigated the ICAM1 expression in anti-ICAM1 CAR-T cell treatment *in vivo*. IHC demonstrated obvious decrease in ICAM1 expression in the ICAM1-CAR-T treatment group (Figures 7A,C). In addition, to evaluate the therapeutic effects of CAR-T cells, IHC analysis of mouse tumor was performed. In the histological analyses of the tumor, we found prominent infiltrate of human T cells in the ICAM1-CAR-T group but not in the control CAR-T or PBS group (Figures 7B,D). Taken together, the above results show that anti-ICAM1 CAR-T cells exhibit excellent antitumor response against TNBC.

## Discussion

Treatment of TNBC has been proven very challenging because of its high invasiveness and recurrence rate and the poor prognosis. CAR-T provides a new promising therapeutic strategy for tumors. Due to the significant breakthrough in the treatment of hematologic malignancies by CAR-T, the application of CAR-T to solid tumors has become a research hotspot in the field of immunotherapy (32). Antigen-specific CAR-T cells recognize tumor-associated antigen *via* an antigen-binding domain. Therefore, an ideal tumor target antigen is crucial for engineering CAR T cells.

Intercellular adhesion molecule-1 is an important transporter that is upregulated in several types of cancers. ICAM1-targeting therapy has potential application values in the treatment of some malignancies, including thyroid cancer, pancreatic cancer, and gastric carcinoma (20, 33, 34). In this study, we analyzed the expression of ICAM1 in TNBC based on the UALCAN database. We found that ICAM1 mRNA expression was higher in TNBC compared with the adjacent normal breast tissue and other types of breast cancer. In addition, immunohistochemical analyses revealed that ICAM1 expression was absent or weak in non-TNBC tissues but could be detected at high expression level in TNBC tissues. In previous studies, Guo and his colleagues provided evidence that the *in vitro* TNBC cell-ICAM1 antibody binding force on live cells correlated with the *in vivo* tumor accumulation and therapeutic efficacy of ICAM1 antibody-directed liposomes (25). We examined several cell lines of breast cancer and found that MDA-MB-231 and MDA-MB-468 had the highest expression level of ICAM1. Therefore, we constructed ICAM1-specific CAR-T cells to investigate whether CAR-T cells had antitumor activity on TNBC *in vitro* and *in vivo*. We observed that ICAM1-specific CAR-T cells could effectively kill ICAM1-positive TNBC cells *in vitro*. By establishing a mouse xenograft model, we found that ICAM1-specific CAR-T caused regression of established cancer and prolonged survival. In addition, HER2-amplified luminal breast cancer cell line SKBR3 cells (ICAM1 negative) were selected as control cells in our study. We have proved that ICAM1-specific CAR-T cells were not able to kill SKBR3 cells *in vitro* and *in vivo*, indicating that ICAM1 would be an ideal tumor-associated antigen target for CAR-T cells to treat TNBC.

It is noteworthy that ICAM1 expression could be induced by proinflammatory cytokines, in breast cancer cell lines and normal breast epithelial cells (35). Cytokines secreted by CAR-T cells are key indicators of their antitumor activity (36). Thus, cytokine signaling pathways can induce ICAM1 expression in neighboring healthy cells, which leads to limited therapeutic efficacy or life-threatening toxicity. Some strategies to further improve the ICAM1-specific CAR-T cells are to lower the antigenic affinity of CARs and increase the selectivity of T cells only for targets that are highly expressing antigens and not for the weakly expressing normal cells. Decreasing the scFv affinity (1–100 μM) or using the LFA-1 I domain in the micromolar range (∼10 μM) as extracellular antigen binding domain produced effective tumor elimination in the absence of toxicity and exhibited a significantly higher therapeutic index *in vivo* (33, 35). A previous study has shown that ICAM1 and EGFR are optimal candidates for TNBC complementary targeting and suggested that it may be necessary to develop an ICAM1/EGFR-bispecific CAR-T cell approach to overcome the risk of off-target toxicity (26). In addition, the tri-specific CAR-T cells such as combining ICAM1 with other TNBC surface antigens are constructed to improve the safety and selectivity of CAR-T cells in TNBC therapy.

In conclusion, we confirmed that ICAM1-specific CAR-T cells were able to efficiently recognize ICAM1 expressing TNBC cells, and they reduced the TNBC tumor growth effectively both *in vitro* and *in vivo*. Therefore, we provide a framework for the adaptation of CAR-T cells targeting ICAM1 as a new treatment modality for TNBC.

## Data Availability Statement

All datasets presented in this study are included in the article/Supplementary Material.

## Ethics Statement

The animal study was reviewed and approved by the Biomedical Ethics Committee of West China Hospital of Sichuan University.

## Author Contributions

HW and ZWa contributed to the concept development and study design, performed the majority of experiments, and wrote the article. YK, ZWu, SZ, and ZZ performed the laboratory experiments, participated in figure preparation, and revised the article. HL, MZ, and NZ performed the laboratory experiments and data analysis. CL, WG, and CN revised the article. HY and AT conceived and designed the study. All authors have reviewed and approved the manuscript.

## Conflict of Interest

NZ was employed by West China-Frontier Pharma Tech Co., Ltd. The remaining authors declare that the research was conducted in the absence of any commercial or financial relationships that could be construed as a potential conflict of interest.

## Footnotes

1 http://ualcan.path.uab.edu/index.html

2 https://portals.broadinstitute.org/ccle/home

3 http://chopchop.cbu.uib.no
