# Supplementary material for: Intercellular Adhesion Molecule-1 as Target for CAR-T-Cell Therapy of Triple-Negative Breast Cancer
Source: Front Immunol. 2020 Sep 23;11:573823. doi: 10.3389/fimmu.2020.573823 (PMC7539633; doi:10.3389/fimmu.2020.573823)
Supplement: Supplementary file 1 [file Data_Sheet_1.PDF]

Supplementary Material

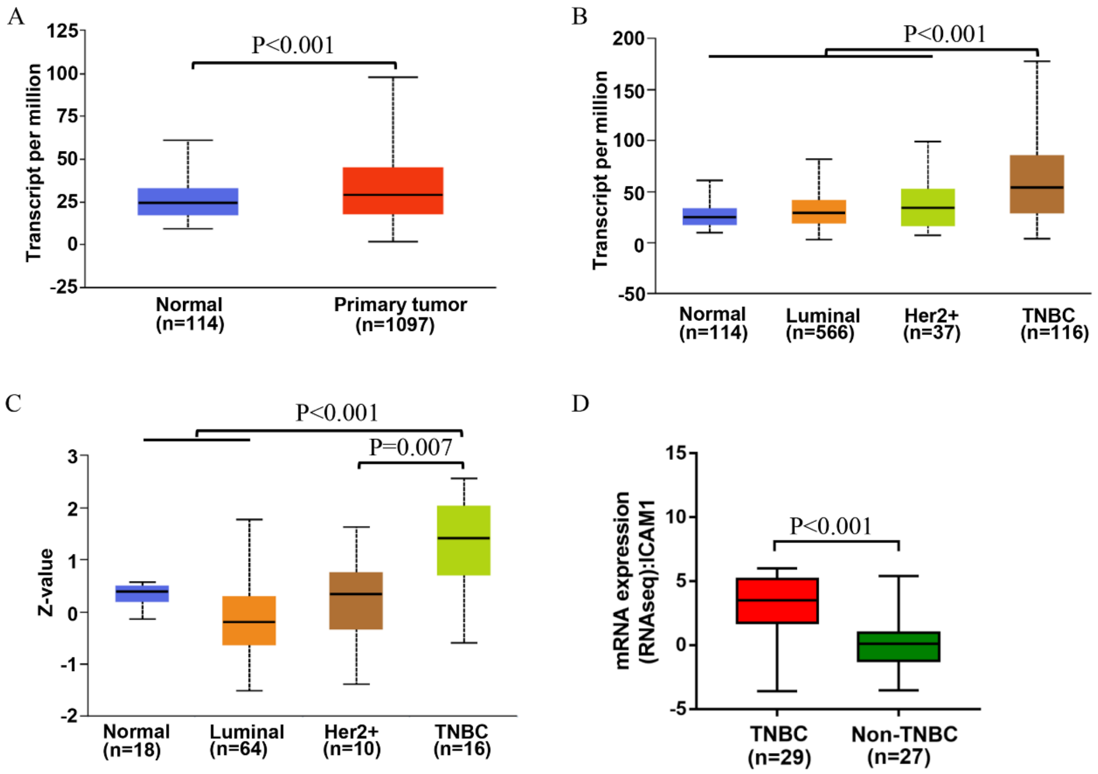

Supplementary Figure 1. Box-whisker plots showing the expression of ICAM1 in breast invasive carcinoma samples (BRCA). **(A)** Boxplot showing relative expression of ICAM1 in normal and BRCA samples. **(B)** Boxplot showing relative expression of ICAM1 in normal, luminal, HER2 positive and TNBC patients. **(C)** Protein expression of ICAM1 in normal, luminal, HER2 positive and TNBC patients. Z-values represent standard deviations from the median across samples for the given cancer type. Log2 spectral count ratio values from CPTAC were first normalized within each sample profile, then normalized across samples. **(D)** Expression analysis of ICAM1 mRNA in breast cancer cell lines based on CCLE database.

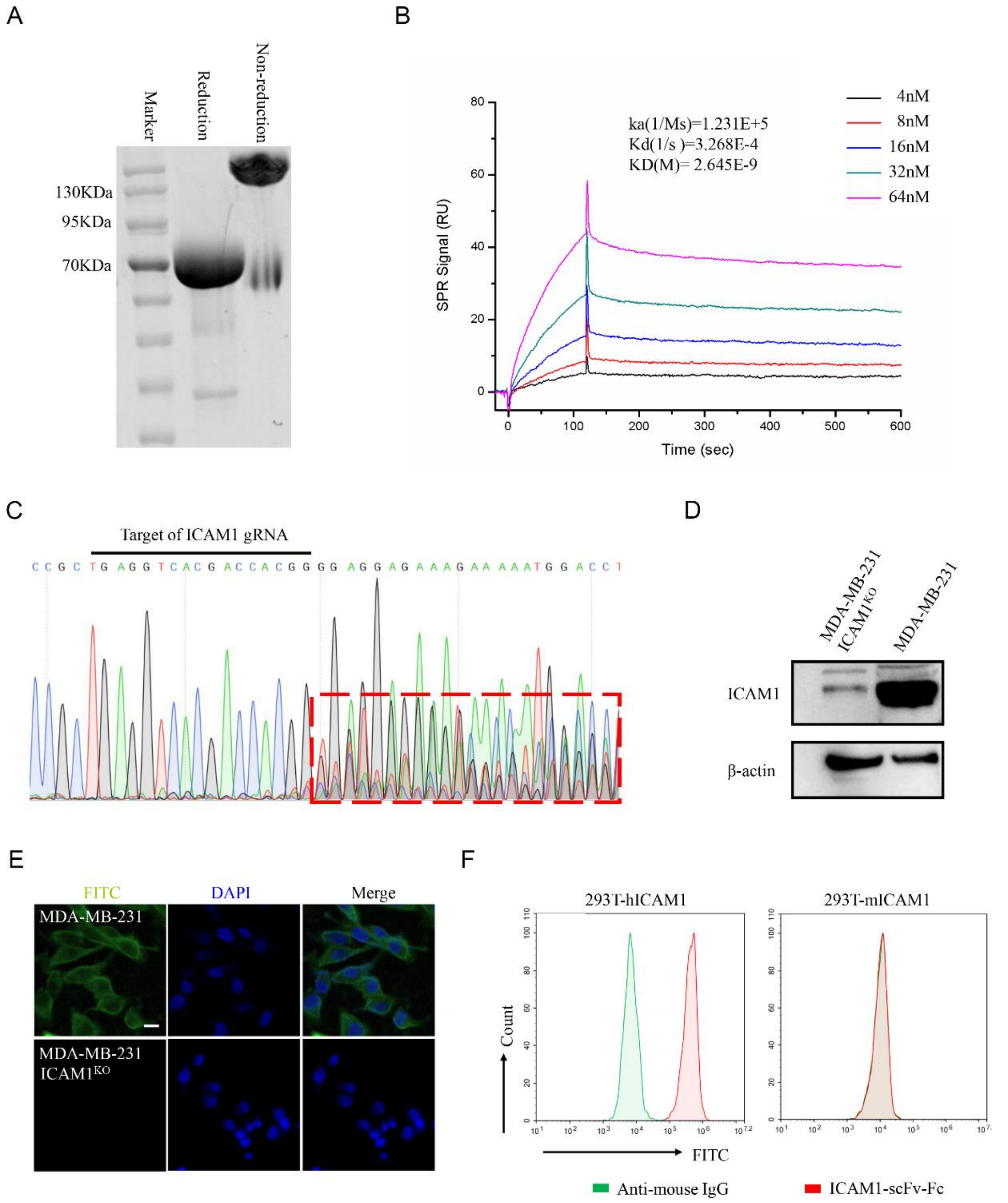

Supplementary Figure 2. Identification and affinity purification of ICAM1-specific scFv proteins. **(A)** SDS-PAGE results for purified mG2-scFv-Fc. **(B)** The affinity of mG2-scFv-Fc binding to ICAM1(ECD) protein was determined on a Biacore X100 instrument. RU, resonance units. **(C)** Sequencing results for MDA-MB-231 cells, 5 days after transduction with lentivirus carrying ICAM1-targeted gRNA and Cas9. **(D)** Expression of ICAM1 in MDA-MB-231 and MDA-MB-231 ICAM1^{KO} cell surface were analyzed by western blotting. **(E)** IF staining with mG2-scFv-Fc in FACS-sorted ICAM1^{KO} MDA-MB-231 cells and in wild type of MDA-MB-231 cells. Scale bar, 50 μm. **(F)** The specificity of mG2-scFc to 293T cells stably transduced to express human and mouse ICAM1 was determined by FACS.

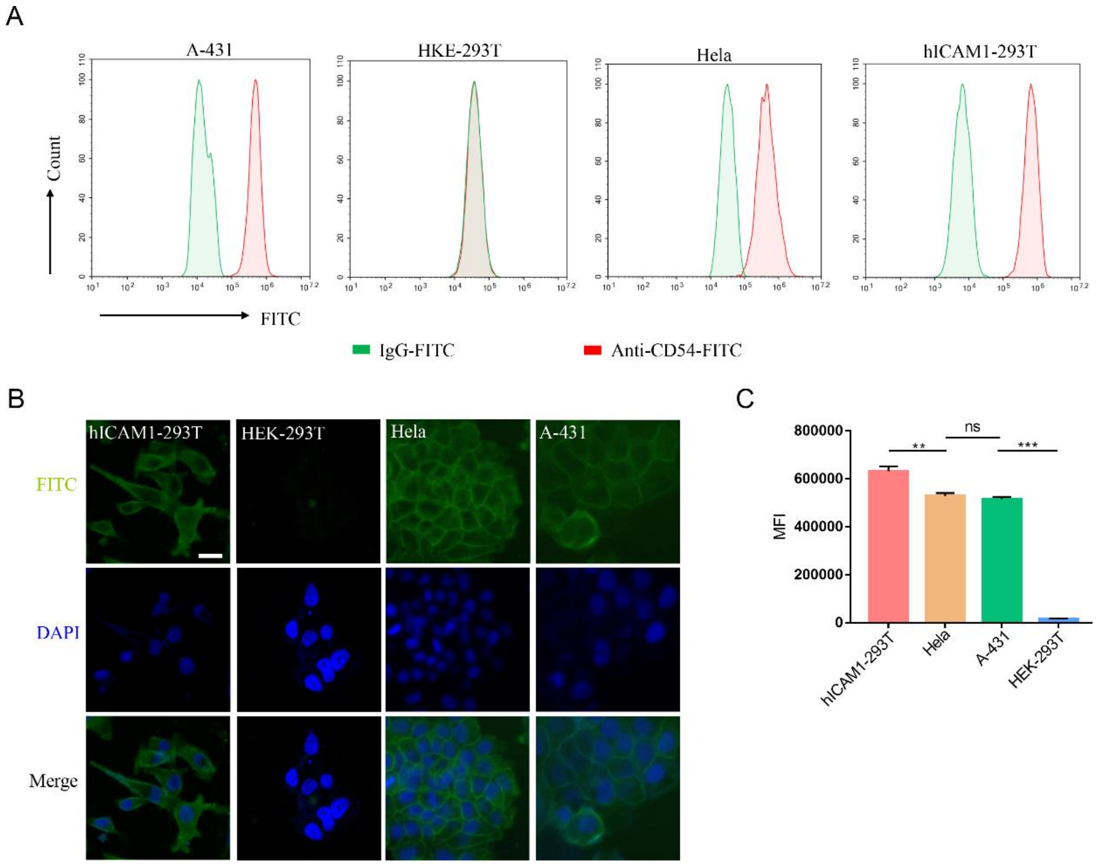

Supplementary Figure 3. The ability of mG2-scFv to bind to endogenous ICAM1 proteins in human tumor cell lines. **(A)** Expression of ICAM1 in Hela, A-431, hICAM1-293T and HEK-293T cell surface were evaluated by FACS. **(B)** IF staining with mG2-scFv-Fc in Hela, A-431, hICAM1-293T and HEK-293T cells. Scale bars, 50 μm. (C) Quantification of ICAM1 staining intensities in various cell lines. Unpaired two-tailed Student's t tests were applied. $^{**}p<0.01$, $^{***}p < 0.001$.

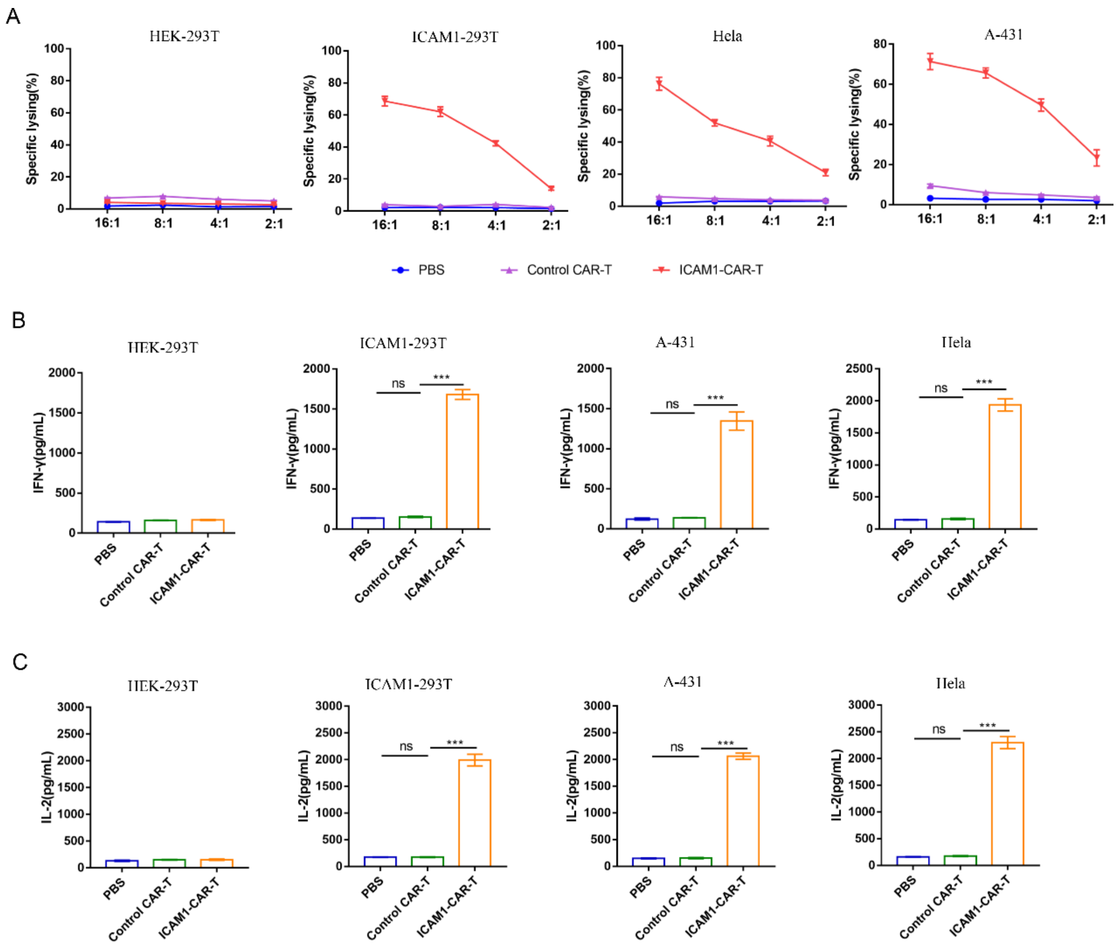

Supplementary Figure 4. ICAM1-specific CAR-T cells target ICAM1-positive cell lines killing in vitro. **(A)** ^{51}Cr-release assay to measure the cytotoxicity of CAR-T cells against Hela, A-431, ICAM1-293T and HEK-293T cells at E:T ratios of 2:1, 4:1, 8:1 and 16:1. **(B)** and **(C)** The concentrations of IL-2 and IFN-γ released by ICAM1-specific CAR-T cells, control CAR-T cells and PBS after co-culture with target cells 24h at an E:T ratio of 4:1. Each experiment was repeated three times. Unpaired two-tailed Student's t tests were applied. ***$p < 0.001$.
