# Supplementary material for: Intercellular Adhesion Molecule-1 as Target for CAR-T-Cell Therapy of Triple-Negative Breast Cancer
Source: Front Immunol. 2020 Sep 23;11:573823. doi: 10.3389/fimmu.2020.573823 (PMC7539633; doi:10.3389/fimmu.2020.573823)
Supplement: Supplementary file 2 [file Table_1.DOCX]

| Variables | N of Samples | ICAM1 intensity score  (Mean±SEM) |
| --- | --- | --- |
| -/-/- | 90 | 117.4±7.318 |
| +/-/+ | 5 | 19±1.549 |
| -/+/+ | 3 | 25.67±1.856 |
| -/-/+ | 3 | 31.33±1.764 |
| +/-/- | 17 | 41.29±5.715 |
| +/+/- | 24 | 47.67±5.088 |
| +/+/+ | 33 | 54±6.88 |

**Table S1**. The constituent ratio of different ICAM1 expression levels under different clinicopathologic variables in 90 TNBC patients and 85 non-TNBC patients.
